# Stress Level Detection Based on the Capacitive Electrocardiogram Signals of Driving Subjects

**DOI:** 10.3390/s23229158

**Published:** 2023-11-14

**Authors:** Tamara Škorić

**Affiliations:** Faculty of Technical Science, University of Novi Sad, 21000 Novi Sad, Serbia; ceranic@uns.ac.rs

**Keywords:** unobtrusive health monitoring system, machine-learning model for stress level detection, cECG filter

## Abstract

The automotive industry and scientific community are making efforts to develop innovative solutions that would increase successful driver performance in preventing crashes caused by drivers’ health and concentration. High stress is one of the causes of impaired driver performance. This study investigates the ability to classify different stress levels based on capacitive electrocardiogram (cECG) recorded during driving by unobtrusive acquisition systems with different hardware implementations. The proposed machine-learning model extracted only four features, based on the detection of the R peak, which is the most reliably detected characteristic point even in inferior quality cECG. Another criterion for selecting the features is their low computational complexity, which enables real-time application. The proposed method was validated on three open data sets recorded during driving: electrocardiogram (ECG) recorded by electrodes with direct skin contact (high quality); cECG recorded without direct skin contact through clothes by electrodes built into a portable multi-modal cushion (middle quality); and cECG recorded through the clothes without direct skin contact by electrodes built into a car seat (lowest quality). The proposed model achieved a high accuracy of 100% for high-quality ECG, 96.67% for middle-quality cECG, and 98.08% for the lower-quality cECG.

## 1. Introduction

Almost 20 thousand people died in traffic accidents in 2021 in the European Union [1], and the number of deaths per million inhabitants in Serbia is higher, reaching 30% [2]. The European Union has set itself the goal of reducing the number of traffic fatalities by 50% by 2030, and it is expected that zero traffic fatalities (‘Vision Zero’) will be reached by 2050 [1]. Drowsiness, the growth of licensed older drivers, and health problems have been identified as the main causes of traffic accidents. Artificial intelligence (AI) has revolutionized the development of autonomous cars, but mass production is in the distant future. Application in everyday conditions is particularly difficult due to several open questions, such as car-to-car communication, complete amendment of legal regulations, etc. A transitional solution is semi-autonomous cars with a system for monitoring the driver’s health.

From July 2024, all new-type registered motor vehicles sold in the EU must comply with the updated General Safety Regulations, which require a range of mandatory advanced safety services [3]. The automotive industry as well as the scientific community are making efforts to develop innovative solutions that would increase the safety of drivers and children in cars, as well as comfort and services that facilitate driving itself [4,5,6,7].

The scientific community is focused on developing a health monitoring system that does not require medical assistance and ensures comfortable and undisturbed driving. To provide a comfortable environment and enable daily application, these systems must not require the placement of electrodes or cuffs after applying the gel directly to the subject’s body, in contrast to clinical practice. As a result, the signal quality is significantly lower compared to signals recorded in controlled clinical conditions, and their processing represents a current scientific challenge. Jointly, the scientific community and the automotive industry proposed multisensor systems built into a car seat for recording the ECG of the Mercedes S class (2009; explained in detail in [8]), into the steering wheel (measurement of blood oxygen saturation level (SpO2) and body temperature) of the Mercedes S class (2010, explained in detail in [9]), into of car seat of a Ford car (2011, explained in detail in [10]) and, finally, a proposal from 2023 for a portable cushion with built-in capacitive electrodes [11]. There is also a health monitoring system based on high-tech cameras [12,13,14], but it is not a favorable choice from the aspect of violating the privacy of the driver and passengers and taking longer to process images.

Successful driver performance in preventing vehicle crashes determines drivers’ general health and concentration levels that may be impaired in case of high-stress exposure. The development of gastrointestinal and musculoskeletal disorders for professional drivers is also considered a consequence of chronic stress, and the development of posttraumatic stress disorder is also reported as a common chronic mental health issue in ambulance and bus drivers, respectively [15,16]. One of the open questions is how stress during driving influences driver health, especially in older drivers, and pacemaker-built-in drivers. In the state-of-the-art literature, there is evidence to support a pathophysiological basis for the increased incidence of arrhythmias during driving, but there is a lack of convincing causal evidence linking driving activity to arrhythmia generation [17].

A detailed review of the literature (published from 1990 to 2007) related to the development of algorithms for the detection of stress levels was presented in [18]. The presented algorithms are based on feature extraction of direct-to-skin contact measurement ECG, electrodermal activity, respiratory response, electrodermal activity, and blood pressure. In the literature published from 2007 to 2022, traditional machine learning models based on feature extraction and deep transfer learning techniques were proposed for stress level detection based on ECG by recorded direct skin contact. A traditional machine learning model based on 14 features extracted by characteristics point of ECG for detection of three stress levels (with an achieved accuracy of 88.24%) was proposed in [19]. M. Amin et al. [20] proposed the use of deep learning techniques with input as scalogram images (1D ECG signals were transformed into 2D time-frequency images (scalograms)) and achieved an accuracy of 98.11% for stress level detection. In [21], the authors proposed the use of a multimodal fusion model based on convolutional neural networks (CNN) and long short-term memory (LSTM) to fuse the ECG, vehicle data and contextual data to jointly learn for stress level detection during driving (achieved average accuracy was 92.8%). M. Rastgoo et al. [18] highlight the challenges that may arise in the application of algorithms in the real-world driving scenario with the use of unobtrusive acquisition systems such as data quality and time-consuming data analysis of high dimensionality. Also, J. Wang et al. (2020) emphasized that there are no scientific studies dealing with the detection of driver’s illness on non-contact recorded signals while driving [22].

A new challenge facing the scientific community is the processing of inferior quality signals recorded in everyday conditions without the supervision of medical experts. Inferior quality is a consequence of limiting factors such as measurement without direct skin contact with the driver (recording over clothes made of different materials), the dominant presence of movements during driving, as well as the higher sensitivity of capacitive electrodes to noise and the capacitive electrodes being more affected by motion artifacts than the conductive one [11]. An cECG time series recorded during driving has an extremely low amplitude, approximately 1 mV, which is up to several tens of times lower intensity than the signals recorded in controlled clinical conditions [23], with the predominant presence of movement artifacts (on average, 30% of the time series recorded during driving is useless [24]). Despite such low quality, it has been shown that it is possible to reliably detect the R peak (the local maximum of the ECG signal), based on which the heart rate (HR) is estimated [10]. A higher cECG amplitude level was achieved by proposed different hardware realization proposed in [11].

This paper aims to develop a machine learning model for the detection of stress levels based on capacitive electrocardiogram (c)ECG recorded in an unobtrusive acquisition system that could be applicable in daily driving. The basic idea of the proposed method is to select a feature list based only on characteristic points of cECG that are possibly reliably estimated regardless of the cECG quality level, recorded during driving. The feature list is also constrained by feature selections that are not computationally demanding to enable the application of this model in real-time conditions. We expect that the proposed model will achieve high performance regardless of the quality of datasets and hardware realization acquisition system. For the development and validation of model, we used three open database sets: (1) ECG recorded by direct skin contact during driving (Database 1, the highest quality), (2) cECG recorded non-direct skin contact through clothes by capacitive electrodes built into a portable multi-modal cushion during driving (Database 2, modest quality), and (3) cECG recorded through the clothes no-direct skin contact by capacitive electrodes built into a car seat during driving (Database 3, the lowest quality). In each experiment, drivers were exposed to different stress levels: city driving (higher stress level) and highway driving (lower stress level) in Database 1, driving with distraction (by talking, or obligating driver’s moving according to predefined protocol) as higher stress level and driving without distraction as lower stress level in Database 2, and finally city driving (higher stress level) and open roads driving (lower stress level) in Database 3.

## 2. Materials and Methods

### 2.1. Materials

The proposed method is validated on three open datasets recording during driving: ECG recorded by electrodes with direct contact with skin; cECG recorded without direct skin contact through clothes by capacitive electrodes built into a portable multi-modal cushion; and cECG recorded through the clothes without direct skin contact by capacitive electrodes built into a car seat. All drivers that participated in the experiment were healthy volunteers.

#### 2.1.1. ECG Recorded by Direct Skin Contact during Driving (Database 1)

The acquisition system consisted of an electrocardiogram (ECG), electromyogram, skin conductivity (electrodermal activation, and galvanic skin response), and respiration sensors [25]. The FlexComp analog-to-digital converter (sampling frequency is 496 Hz) was connected to an embedded computer.

ECG electrodes were directly placed on the volunteer’s skin. The skin was prepared by using alcohol as a cleanser and Electro-Trace pre-gelled before ECG electrodes were placed [26]. Modified lead II configuration was used to minimize motion artifacts and maximize the amplitude of the R waves.

In the experiment, 24 healthy volunteers were involved. Driver protocol consisted of a driving period of rest, highway, and city driving in the Boston area [25]. The total duration, including rest periods, was from 50 min to 1.5 h, depending on traffic conditions. The stress level metric was estimated based on a driver questionnaire and a score derived from observable events and actions coded from video recordings. Obtained results have shown that a high level of stress was achieved for driving in city environments, and a medium level in driving highway environments. An open data set provided data recorded on 16 out of 24 drivers with complete recorded data and metrics for stress levels. The mean length [samples] ± SD of cECG is 219,590 ± 40,860 samples.

The database with all experimental recordings is publicly available in [27], and the experiment is described in detail in reference [25]. All volunteers gave written consent. For a clearer presentation of the results in tables and figures, these data are marked with Database 1.

#### 2.1.2. cECG Recorded by Non-Direct Skin Contact through Clothes by Capacitive Electrodes Built into a Portable Multi-Modal Cushion during Driving (Database 2)

The acquisition system consists of four sensor units that include a reflective photoplethysmography sensing unit, a magnetic induction measurement sensing unit, an accelerometer, and one electrode for cECG built into a portable multi-modal cushion. The sampling frequency for each modality is 128 Hz. The cECG electrode is realized using a shielded high-impedance operational amplifier, OPA140 (Texas Instruments, Dallas, TX, USA). The amplifier output by coaxial cable is led to the controller box, where the ECG leads were obtained. Detailed block schemes and photographs are given in [11].

In the experiment, 20 healthy subjects (18 males and 2 females, aged 25.9 ± 8.53 years) were used driving simulators for around 25 min [11]. The open-source simulator CARLA was used for simulating driving, with the selected environment “Town04” [11]. The driving was simpler than the driving conditions in real-world conditions because the drivers were not obliged to follow the traffic rules.

The experiment consisted of four stages: stage 1 (ST1)-driving in a salient environment (without talking with the driver), stage 2 (ST2)-movement of the driver during driving according to protocol, stage 3 (ST3)-distracting the driver with a conversation that simulates the presence of passengers, and stage 4 (ST4) siting without driving and talking [11]. The mean length of cECG, expressed in samples, is 722,332.

The database with all experimental recordings is publicly available in [28], and the experiment is described in detail in reference [11]. All volunteers gave written consent.

The study protocol was a by the ethics committee of RWTH Aachen University Hospital (EK 183/22). For a clearer presentation of the results in tables and figures, these data are marked with Database 2.

#### 2.1.3. cECG Recorded through the Clothes No-Direct Skin Contact by Capacitive Electrodes Built into a Car Seat during Driving (Database 3)

The acquisition system was based on 6 capacitive electrodes built into the car seat. The electrodes were arranged on three levels per two capacitive electrodes in the upper part of the car seat. Three electrodes with the most reliable measurements were manually selected [29]. The sampling frequency was 200 Hz. A detailed block scheme and photograph was given in [10,29].

In the experiment, 6 male volunteers (mean aged 39.8 ± 26.2 years) were involved. cECG time series were recorded during driving in the city (about 2 h of recording), on the highway (about 8.8 h of recording), and in the polygon in Belgium (about 2.5 h of recording) in real-world conditions [29]. The total number of measurements was 31 measurements, consisted of three cECG time series (marked by cECG_1_ = Electrode_1_ − Electrode_2_, cECG_2_ = Electrode_2_ − Electrode_3_, and cECG_3_ = Electrode_3_ − Electrode_1_), as well as the reference ECG signal. The mean length of cECG, expressed in samples, is 1556.93 per channel. The estimated level of signal-to-noise ratio SNR was −40.01 ± 33.64 dB, for cECG_1_ and −30.99 ± 23.39 dB for cECG_2_ during driving [24]. The negative value of SNR was a consequence of very high amplitude values of the coarse artifacts if compared to the useful parts of the signal (medical experts labeled useful and useless parts of the cECG time series). The high standard deviation is a consequence of the different amounts of coarse artifacts in the cECG time series in Database 3.

The database with all experimental recordings is publicly available in [28], and the experiment is described in detail in reference [29]. All volunteers gave written consent. For a clearer presentation of the results in tables and figures, these data are marked with Database 3.

The mean absolute amplitude value of (c)ECG time series, number of participants, and sampling frequency ECG for Database 1 (direct skin contact), Database 2 (non-direct contacts by capacitive electrodes built-in portable cushion), and Database 3 (non-direct contacts by capacitive electrodes built-in car seat) is shown in Table 1.

It is noticeable that the hardware solution for cECG acquisition (Database 2), proposed in [11], achieved higher amplitude intensity during driving compared to Database 3. Although the amplitude intensity of Database 1 (recorded with direct skin contact) is comparable to the amplitude value achieved in Database 3, the signal quality is significantly better due to fewer motion artifacts as a consequence of direct skin contact. Table 1 also shows that the sampling frequency is different for each dataset.

### 2.2. The Proposed Method

#### 2.2.1. Framework of the Proposed Method

In the recent literature (e.g., [19,26,30]), stress level detection was determined based on parameters extractions from the ECG signal, muscle activity or electromyogram (EMG), skin conductance or electrodermal activity (EDA), and respiration (RSP) recorded by direct contact with the skin (high-quality recordings).

A new challenge facing the science community is the development of methods for estimation of the stress level based on unobtrusive recorded signals, through clothing, so that these acquisition systems can be candidates for everyday use in the automotive industry. In the first place, these signals are characterized by an inferior quality compared to the gold standard recordings in controlled clinical conditions, especially due to the significant presence of motion artifacts and recordings without direct skin contact. Inferior quality and the dominant presence of motion artifacts may contribute to the unreliably detection of P, Q, R, S, and T points (PQRST complex) in unobtrusively recorded cECG time series. In [31], it is emphasized that reliable identification of P and T waves in cECG is not possible, mostly due to the presence of movement artifacts.

**Figure 1:**
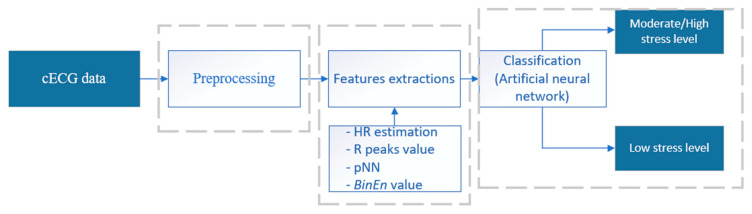
Block scheme of procedure for stress classification during driving. Feature list: heart rate (*HR*), R peaks value (local maximum in ECG), and percentage of differences between adjacent normal cardiac intervals (*pNN*) and binarized approximate entropy (*BinEn*).

To enable application in pervasive environments, in the study, we propose a novel machine learning (ML) model based on the extraction of only four features that request only R-peak detection (the local maximum of the ECG signal), which can be the most reliably detected characteristic point in inferior quality cECG time series [10]. As shown in Figure 1, this method consists of three main parts: preprocessing, feature extraction, and classification step.

The detailed steps of the proposed method are as follows:

1. cECG time series were processed by method design for the reduction of coarse motion artifacts in cECG recorded on moving subjects (explained in detail in [24]);
2. Four feature extraction: heart rate (*HR*), the value of R peak, binarized approximate entropy of cECG time series (*BinEn*), and *pNN* (percentage of differences between adjacent normal cardiac intervals);
3. Different type of machine learning methods has been tested, but the most consistent classifier for stress detection levels for three datasets (Database 1, Database 2, and Database 3) has been shown.

#### 2.2.2. Preprocessing cECG Time Series

We have recently proposed a method for artifact reduction in cECG time series, originally developed for cECG recorded on driving subjects (described in detail in [24]). The preprocessing method was validated on cECG recorded during driving by capacitive electrodes built-in car seat (Database 3). Raw cECG time series were segmented to provide real-time application during driving. Artifact reduction is based on estimated fluctuation around linear trends into segmented time series, to identify the presence of coarse artifacts as a consequence of moving subjects during driving or slow-changing artifacts. All parameters used in the pre-processing step were estimated based on statistical parameters of observed *cECG.*

Briefly, the *cECG* time series is marked with *x*, and samples are denoted by $x_{k},\;k=1,\ldots N$, where N represents the length of the time series. Vector of cumulative sums *Y*(*i*)*, i* = 1, …, *N* is formed following [32]:

(1) $$Y\left(i\right)=\sum_{k=1}^{i}\left[x_{k}\right]-xi=1,\ldots ,N.$$

where the *k*^th^ centralized sample is formed by subtracting the mean value of the time series x from the *k*^th^ sample $x_{k}$.

The vector of cumulative sums *Y*(*i*), i=1,…,N, is divided into non-overlapping segments of length *SL.* $\Upsilon_{j}\left(k\right)\;$ represents segments, where *j* is used for the notation of particular segments $\left(\;j=1,\ldots ,\frac{N}{SL}\right)$ and *k* denotes the samples within a particular segment, k=1,…,SL.

In the next step, from $\Upsilon_{j}\left(k\right),\;$ segments are subtracted the approximated polynomial $p_{j,v}$ of the *v*-th order.

(2) $$\Upsilon_{j,SL}\left(k\right)=\Upsilon_{j}\left(k\right)-p_{j,v}\left(k\right),j=1,\ldots ,\frac{N}{SL},k=1,\ldots ,SL,$$

where

(3) $$p_{j,v}\left(k\right)=a_{v}\cdot \Upsilon_{j}{}^{v}\left(k\right)+a_{v-1}\cdot \Upsilon_{j}{}^{v-1}{\left(k\right)}_{}+\ldots +a_{0},$$

$a_{v},\;a_{v-1},\;a_{0}$ is the polynomial coefficients on a segment, and *v* is thepolynomial order [32].

We used linear polynomials (*v* = 1) as recommended in [32].

Detrended fluctuation analysis function of one segment, $F_{D}{}^{}\left(j\right)$, is calculated as the sum of the square value of the difference between the original value of the time series and the trend of a given segment divided with SL [32]:

(4) $$F_{D}{}^{}\left(j\right)\;=\sqrt{\frac{1}{SL}\cdot \sum_{k=1}^{SL}\left\{\Upsilon_{j,SL}^{2}\left(k\right)\right\}},j=1,\ldots ,\frac{N}{SL}.$$

In the first step, we check the presence of coarse artifacts in particular segment according to established criteria proposed in [24]:

(5) $$\left(\left(\max\left(x\right)-\min\left(x\right)\right)/2\right)-\sqrt{M}>1,\;M=E\left(x^{2}\right)=\frac{1}{N}\sum_{k=1}^{N}x_{k}^{2}.$$

where max(*x*) and min(*x*) are maximum and minimum values of time series *x*, respectively, and *M-* the is the second moment of time series *x*.

If the condition from Equation (5) is fulfilled, we then eliminate observed segments if $F_{D}{}^{}\left(j\right)$ is greater than the threshold $TH_{1}$ [24]:

(6) $$TH_{1}=\left(\sqrt{{\left(\left(\max\left(x\right)-\min\left(x\right)\right)/2\right)}^{2}-M}\right)\cdot C\cdot median\left(F_{D}\right)+\frac{\left(SD\left(F_{D}\right)+SD\left(x\right)\right)}{SD\left(F_{D}\right)\cdot SD\left(x\right)}\cdot C_{1},$$

where $median\left(F_{D}\right)$ is the median value of detrended fluctuation function of all segments in time series, $SD\left(F_{D}\right)$ and $SD\left(x\right)$ are the standard deviation of $F_{D}$ and *x*, respectively, max(*x*), min(*x*)-maximum and minimum value of time series *x*, respectively, *M-* the second moment, C is constant value from the range, $C\in \left\{0.15–0.35\right\}$ $\frac{1}{mV^{2}}$ and $C_{1}=1\;\mathrm{mV}$.

To check if coarse artifacts partially spill over the adjacent segments, adjacent segments are also checked [24]. If the difference between $F_{D}\left(j\right)\;$ values and $F_{D}\left(j+1\right)\;$ or $F_{D}\left(j-1\right)\;$ of adjacent segments is larger than $TH_{2}=\frac{TH_{1}\;}{2}$, this segment is also eliminated. To eliminate small deviation from the linear trend, i.e., slow-changing artifacts, we compare the value of $F_{D}\left(j\right)$ with threshold $TH_{3}=0.1$. To avoid the elimination of the useful part of cECG time series where the signal intensity is very small, such as in the case of Database 3, this step is applied only if the difference between the mean value of $F_{D}$ and $SD\left(F_{D}\right)$ is greater than $TH_{3}$ [24].

#### 2.2.3. Feature Extraction

The selection of features was based on the detection of the R peak, the local maximal ECG signal, as the point of the PQRST complex that we can detect with the highest reliability even if the cECG recording quality is not high, as is the case with Database 2. The list of features consists of only 4 features (R value, heart rate, percentage of differences between adjacent normal-to-normal (NN) intervals, and Binarized entropy of cECG time series), which are not computationally demanding and would enable the use of the proposed ML model in real-time. To the best of the author’s knowledge, R-value and Binarized entropy (*BinEn*) are recognized as features for stress level detection during driving for the first time (a detailed overview of the features used to detect driving stress is presented in [18]).

##### Feature Selection Based on R Peaks

In line with [10], we used open-source ECG analysis (detailed described in [33]) for R peaks detection in cECG. Three features out of four are selected based on R peak: R peak value, heart rate (*HR*) based on the time interval between adjusted R peaks (marked as RRI), following:y

(7) $$HR\;\left[beat/minute\right]=\frac{60}{RRI\;\left[ms\right]}\cdot 1000$$

and percentage of differences between adjacent normal-to-normal (NN) intervals (*pNNx*). NN intervals correspond to RRI intervals after artifact reduction, so preprocessing of cECG is an obligatory step in this case.

*pNNx* is estimated following [34]:

(8) $$pNNx=\frac{number\;of\;\;\left|\Delta NN\right|>x}{total\;number\;of\;\Delta NN}$$

where ΔNN is difference between adjusted *NN* intervals, and *x* is predefined value.

In this study, we selected a value of *x* = 50 ms, according to [34].

##### Binarized Entropy (*BinEn*)

The fourth feature presents binarized entropy of cECG, and also does not require detection of PQRST complex in cECG. Binarized entropy (*BinEn*) [35], as a derivative from approximative entropy (for a detailed explanation, see [36]), is an adapted need of mobile crowdsensing systems.

Binarized entropy (*BinEn*) is performed on differentially coded time series, which significantly speeds up the estimation of entropy value and increases the probability that signals fulfill stationarity conditions. The complexity of *BinEn* is linear, and it is achieved by substituting the number of different real vectors with a number of different binary vectors [35]. This method modification makes *BinEn* applicable in real-time conditions.

A brief recapitulation of *BinEn* for a single data set is below.

In the first step, time series x is binary differentially encoded and split into *m*-sized binary vectors [35]:

$$c=\left\{\begin{array}{c} 0\;\;\;\;\;\;\;x_{i+1}-x_{i}\leq 0\\ 1\;\;\;\;\;\;\;x_{i+1}-x_{i}>0 \end{array}\right\}\;\;\;i=1,\ldots N$$

(9) $$C_{m}^{i}=\left[c_{i},c_{i+\tau },\ldots ,c_{i+\left(m-1\right)\cdot \tau }\;\right],i=1,\ldots ,N-\left(m-1\right)\cdot \tau ,\;$$

where the delay *τ* distances the elements of the vector from each other and *m* is the size of the vectors. The recommended value for parameter *τ* = 1, and $m\left\{1,\;2,\;3,\;4\right\}$ [35]. In the *BinEn*, the vectors are binary, so the number of different vectors is $2^{m}$ and each vector can be assigned a decimal number k [35]:

(10) $$k=\sum_{n=0}^{m-1}c_{i+n\cdot \tau }\cdot 2^{n}.$$

$N_{C}^{\left(m\right)}\;$ represents the number of occurrences of a certain vector series in the observed time series C [35]:

(11) $$\;N_{C}^{\left(m\right)}\left(k\right)=\sum_{i=1}^{N-\left(m-1\right)\cdot \tau }I\left\{\sum_{l=0}^{m-1}c_{i+l\cdot \tau }\cdot 2^{l}=k\right\},\;k=0,\;1,\;\ldots ,\;2^{m}-1.$$

$I\left\{\right\}$—indicator function equal to one if the condition is met, and zero otherwise.

The estimation of the probability mass function of observed vectors in C is equal to:

(12) $${\hat{P}}_{C}^{\left(m\right)}=\frac{N_{C}^{\left(m\right)}\left(k\right)}{N-\left(m-1\right)\cdot \tau }\;,$$

Distance d, between each pair of vectors, is calculated according to the Hamming distance [35]:

(13) $$d\left(C_{m}^{i},\;C_{m}^{j}\right)=\sum_{k=0}^{m-1}c_{i+k\cdot \tau }⊕c_{j+k\cdot \tau }=\sum_{k=0}^{m-1}I\left\{c_{i+k\cdot \tau }\neq c_{j+k\cdot \tau }\right\}\;i,j=1,\ldots ,N-\left(m-1\right)\cdot \tau .\;$$

where ⊕ notes ex-or logic function, and *I* $\left\{.\right\}$ the indicators function. The distance $d\left(C_{m}^{i},\;C_{m}^{j}\right)\;$ between the vectors is a discrete variable that can have one of the m+1 values, that is, $d\left(C_{m}^{i},\;C_{m}^{j}\right)\in \left\{0,1,\ldots m\right\}$.

The matrix of the Hamming distance is denoted by *H*(*m*) [35]. Elements of matrix H are the distance between the vector whose decimal represents *k* and the vector whose decimal represents *n,* ($h_{k\cdot n}^{}$). The probability that vector $C_{m}^{i}$ occurs in C is estimated based on the value in matrix H, which gave information about which vectors are in distance less than *r* from $C_{m}^{i}$, and Equation (11), which gave information about a number of vectors that are at the same distance:

(14) $${\hat{p}}_{k}^{m}\left(r\right)=Pr\left\{d\left(C_{m}^{i},\;C_{m}^{}\right)\leq r\right\}=\frac{1}{N-\left(m-1\right)\cdot \tau }\cdot \overset{2^{m}-1}{\underset{n=0}{\sum }}N_{C}^{\left(m\right)}\left(n\right)\cdot I\left\{h_{k\cdot n}^{\left(m\right)}\leq r\right\}\;=\overset{2^{m}-1}{\underset{n=0}{\sum }}{\hat{P}}_{C}^{\left(m\right)}\left(n\right)\cdot I\left\{h_{k\cdot n}^{\left(m\right)}\leq r\right\}$$

In the next step, the value of summand $\hat{\Phi }$ is calculated as the average of logarithm ${\hat{p}}_{k}^{m}$ [35]:

(15) $${\hat{\Phi }}^{m}\left(r,N,\tau \right)=\frac{1}{N-\left(m-1\right)\cdot \tau }\cdot \sum_{k=0}^{2^{m}-1}N_{C}^{\left(m\right)}\left(k\right)\cdot \ln\left({\hat{p}}_{k}^{m}\left(r\right)\right)\;=\sum_{k=0}^{2^{m}-1}{\hat{P}}_{C}^{\left(m\right)}\left(k\right)\cdot \ln\left({\hat{p}}_{k}^{m}\left(r\right)\right)$$

The final value of *BinEn* is estimated on model of approximate entropy (detail in [36]) [35]:

(16) $$BinEn\;\left(m,r,N,\tau \right)={\hat{\Phi }}^{m}\left(r,N,\tau \right)-{\hat{\Phi }}^{m+1}\left(r,N,\tau \right)$$

#### 2.2.4. ML Algorithms

We used different classes of ML algorithms to investigate their potential in the classification of different stress levels in three datasets, recorded during driving with different levels of quality (c)ECG time series.

K nearest neighbors (KNN), as one of the simpler ML algorithms, makes a classification based on the K of the nearest neighbors [37]. The distance between test and training objects in feature space was measured by Euclidean distances. Based on Euclidean distances, test data is classified into: higher stress level (city driving) and lower stress level (driving in highway conditions) for Database 1, higher stress level (distraction of driver by speaking and self-movement during driving) and lower stress level (driving in silent conditions) for Database 2, and higher stress level (city driving) and lower stress level (driving in highway and training ground) for Database 3. The number of K is selected by rules of thumb, k = $\sqrt{number\;of\;data\;points\;in\;training\;data\;sets}$.

The Support Vector Machine (SVM) is another class of machine learning that we used to investigate the potential in classification for three datasets, independently. SVM is based on statistical learning theory. It maps the input data onto a higher-dimensional feature space, with the idea of finding a hyperplane that separates space perfectly into its two classes [38].

The Artificial Neural Networks (ANN), as one of the most effective machine learning tools [37], is also investigated. We used a two-hidden-layer feed-forward network, where the weight coefficients were estimated in one direction from the input to the output layer without looping back. We used mean-square error as the loss function, and hyperbolic tangent sigmoid function as the activation function. The number of neurons in hidden layers was 20, the learning rate was 0.5, and we used networks with backpropagation training algorithm.

### 2.3. Statistical Analysis

Considering that a small number of healthy volunteers participate in experiments (Table 1), we divided the cECG time series recorded while driving into non-overlapped parts with a duration of 50s. This was made possible by long hours of recordings within each database. The time duration to complete the database is given in Table 1. The total size of the data set was 1098 (Database 1: 254, Database 2: 189 and Database 3: 655) after excluding cECG time series for which we did not have all the labeled data, the cECG time series that after preprocessing did not have satisfactory length for useful part of a signal (special in case Database 3), and part of an experiment that was not the focus of this study (for example, recording was in the rest period of Database 1, or siting without driving and talking in Database 2). In this way, we have provided a database of suitable size for testing and validating ML algorithms. Datasets were normalized by subtracting the mean value of each feature and a division by the standard deviation.

The datasets are imbalanced (Database 1: 72.44% is higher stress class, and 27.56% lower stress class, Database 2: 33.33% is higher stress class, and 66.67% lower stress class, and Database 3: 25.19% is higher stress class, and 74.81% lower stress class), so we opted for balanced accuracy. We also estimated Matthew’s correlation coefficient (MCC) because both classes are equally important, and it summarizes the classifier performances in a single value (from −1 to 1).

Classification performance is calculated according to the following expressions:

(17) $$\mathrm{Balanced}\;\mathrm{Accuracy}=\frac{1}{2}\cdot (\frac{TP}{TP+FN}\;+\frac{TN}{TN+FP})$$

(18) $$\mathrm{Sensitivity}=\frac{TP}{TP+FN}$$

(19) $$\mathrm{Specificity}=\frac{TN}{FP+TN}$$

(20) $$\mathrm{Positive}\;\mathrm{predictive}\;\mathrm{value}\;(\mathrm{PPV})=\frac{TP}{TP+FP}\;$$

(21) $$\mathrm{Negative}\;\mathrm{predictive}\;\mathrm{value}\;(\mathrm{NPV})=\frac{TN}{TN+FN}.$$

(22) $$\mathrm{Matthew}’s\;\mathrm{correlation}\;\mathrm{coefficient}\;(\mathrm{MCC})=\frac{TN\cdot TP-FN\cdot FP}{\sqrt{\left(TP+FP\right)\cdot \left(TP+FN\right)\cdot \left(TN+FP\right)\cdot \left(TN+FN\right)}}$$

The classification performance was tested in the context of quantifying the success of the detection of the stress level. We distinguish between two classes: higher stress level (city driving) and lower stress level (driving in highway conditions) for Database 1, higher stress level (distraction of driver by speaking and self-movement during driving) and lower stress level (driving in silent conditions) for Database 2, and higher stress level (city driving) and lower stress level (driving in highway and training ground) for Database 3.

In this context, TP denotes the number of cases correctly identified as higher stress level, FP denotes the number of cases incorrectly identified as higher stress level, TN denotes the number of cases correctly identified as lower stress level, FN denotes the number of cases incorrectly identified lower stress level for each database sets. Training and test datasets consisted of cECG segments of different groups of subjects. In both training and test datasets, both classes (high and low stress levels) are present in an imbalanced proportion that is characteristic of these databases. Cross-validation is used to evaluate and validate the performance of machine learning models.

Some of the illustrative results are presented as graphs showing mean ± standard deviation. Statistical significance between observed groups was checked by *t*-test for paired samples in MATLAB R2019a. We used a significance level *p* < 0.01 for all compared groups.

## 3. Results

### 3.1. Preprocessing Results

In this study, we examined the efficiency of coarse artifacts reduction in cECG recorded during driving by preprocessing method described in detail in [24]. This method was validated and developed in cECG from Database 3, so preprocessing results for those datasets are not included in this study.

**Figure 2:**
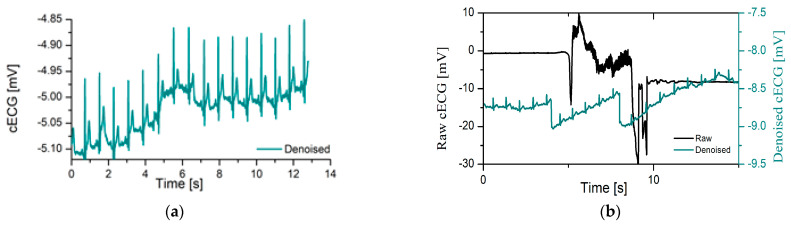
Raw and denoised cECG time series recorded during ST 1 and ST 2 of experiment, Database 2: (**a**) denoised (dark green line) cECG time series recorded during driving in the salient environment (without talking with driver); (**b**) raw (black line) and denoised (dark green line) cECG time series recorded during moving driver according to predefined protocol (ST 2, Database 2).

Figure 2 shows raw and denoised cECG from Database 2 (cECG recorded by electrode built-in portable multi-modal cushion, non-direct skin contact) for a different stage of experiments. Figure 2a represents cECG time series recorded while driving in silence (without any kind of disturbance while driving). Raw cECG time series and denoised cECG recorded perfectly overlapped, as a consequence of non-fulfillment of the conditions in Equation (5), i.e., coarse artifacts are not detected, which is in line with the expectation (in ST1 drivers should not move according to predefined protocols). Due to the impossibility of distinguishing denoised and raw cECG time series, the raw signal display is skipped.

Figure 2b shows raw and denoised cECG time series during ST 2; in this experiment, the stage subject moved while driving according to a predefined protocol. Coarse artifacts were observed in the period from 5 to 10 s. Denoised cECG was shown by the dark green line, and the time axis was scaled.

**Figure 3:**
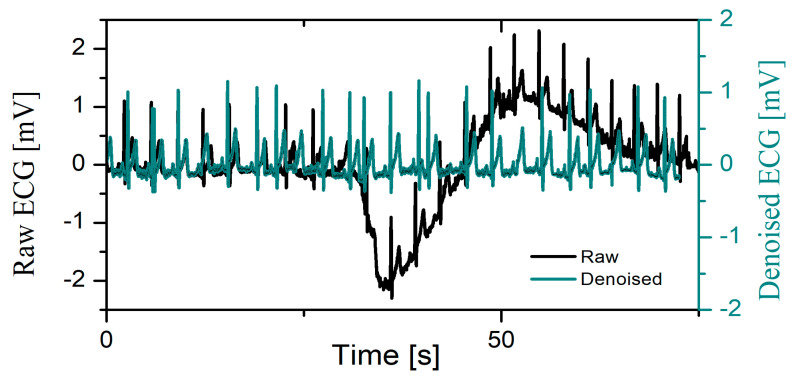
Example of raw (black line) and denoised ECG (dark green) recorded by direct driver’s skin contact during driving.

Visual inspection reveals that the number of ECGs which contain coarse artifacts in Database 1 (direct skin contact measurement) is lower in comparison to cECG records in Database 2 and Database 3 (non-direct skin contact measurement, recording through clothes). Figure 3 represents a rare example of a raw (black line) time series and its denoised ECG time series (dark green line) recorded by direct contact with the skin (Database 1) in the same way as it is recorded in the control clinical condition. The time axis of denoised ECG is scaled after the elimination of coarse artifacts.

**Figure 4:**
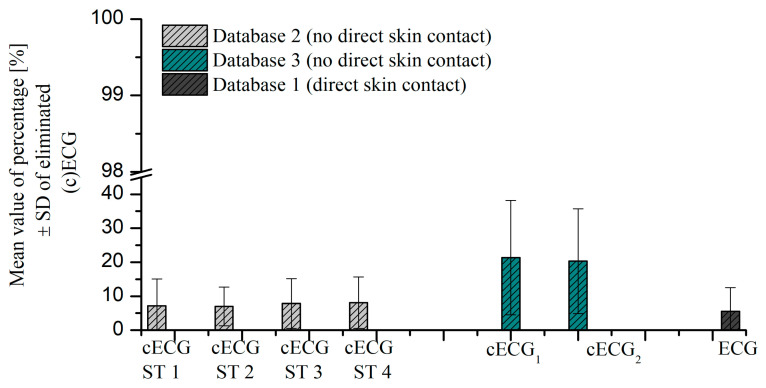
Comparison of the mean value of percentage [%] of eliminated samples ± SD for (c)ECG in Database 1 (marked by the dark gray bar), Database 2 (marked by the light gray bar), and Database 3 (marked by the dark green bar).

We compared the percentage of eliminated coarse and slow-change artifacts in ratio to the length of time series for Database 1 (direct skin contact), Database 2 (non-direct skin contact), and Database 3 (non-direct skin contact). Figure 4 shows the mean value of the eliminated samples ± SD percentage. The lowest value is achieved for Database 1 (direct skin contact), which is in accordance with the high quality of recorded time series by the same acquisition system as used in clinical conditions. The highest percentage of eliminated samples is noted for Database 3 (no direct skin contact, the capacitive electrode was built-in car seat), which is in line with results published in [24]. There is no significant difference between the percentage of eliminated samples in a different experiment stage in Database 2 (no direct skin contact, capacitive electrodes were built in portable multi-modal cushion).

### 3.2. Features Selection Results

**Figure 5:**
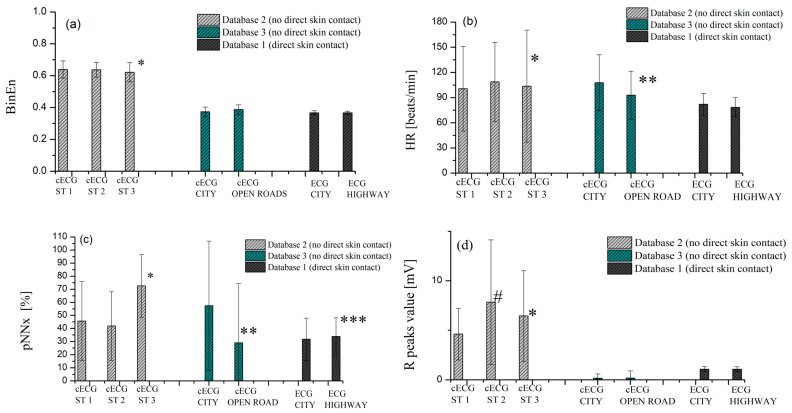
Comparative presentation of the mean ± standard deviation of (**a**) *BinEn*, (**b**) *HR*, (**c**) *pNNx* and (**d**) R peaks value of cECG from Database 1, Database 2, and Database 3. Statistical significance is observed between *cECG* recorded in ST 1 and *cECG* recorded in ST 3 (marked *) for *BinEn*, *HR*, *pNNx*, and R peak values (Database 2). Statistical significance is observed between *cECG* recorded in ST 1 and *cECG* recorded in ST 2 (marked #) only for R peaks value (Database 2). In case of driving in the city and open roads (highways and polygon), there is statistical significance for *HR* and *pNNx* (marked by **). Statistical significance was observed only for *pNNx* in case driving in the city and driving on the highways (marked by ***).

The acquisition of (c)ECG time series in Database 1, Database 2, and Database 3 differs by the hardware implementation of ECG measuring equipment, as well as the method of measurement (with and without direct skin contact), but also by different causes of stress during driving. In the state-of-the-art literature [25], it was observed that a higher level of stress is caused by driving in the city compared to driving on the highway (validation was performed on Database 1 and a detailed assessment of stress metrics is given in [25]). Database 3 was made to distinguish between driving in the city and open roads (including driving on highways and polygons). In Database 2, there are three groups of cECG signals recorded in different driving conditions: driving in the salient environment (ST 1), driving during movement driver according to the predefined protocol (ST 2), and distracting the driver with a conversation that simulates the presence of passengers (ST 3). A driving simulator was used in the experiment, while cECG signals from Database 1 and Database 3 were recorded in real conditions. Figure 5a–d. represents the mean ± standard deviation of four features, extracted based on cECG from Database 1, Database 2, and Database 3.

Statistical significance in all four features (*BinEn*, *HR*, *pNNx*, and *R* values) was observed between driving without driver distraction, in silence (ST 1), and driving simulating conversations with passengers (ST 3). In the case of ST 1 and ST 2 (movement of drivers during driving), statistical significance was only observed at the R peak value. This implies that the distraction of the driver by talking to the passengers has a significant effect on the changes in vital parameters, which can be recorded by (c)ECG.

In the case of driving in the city and on the open road (Database 3), statistical significance was observed only for *HR* and *pNNx*. In the case of Database 1, statistical significance between driving in the city and on the highway was observed only at *pNNx*. Based on the comparative presentation of Figure 5a–d, we can note that different types of stress during driving influence differences in selected features.

Statistical significance between observed groups was checked by *t*-test for paired samples in MATLAB R2019. We used a significance level of *p* < 0.01 for all compared groups.

Figure 5d reveals that the mean value of R peaks is higher for cECG from Database 2, which is a consequence of achieved higher amplitude intensity by new hardware proposals. In this context, we cannot compare the value of R peaks for each dataset, because the intensity of the measured signal is not the same for different hardware solutions (Table 1). The classification was performed for each database individually.

### 3.3. Stress Level Detection by Proposed ML Models

Table 2, Table 3 and Table 4 present classifier (kNN, SVM, and ANN) performances for Database 1, Database 2, and Database 3, respectively. SVM achieved the best performance in the case of Database 1 and Database 2 (marked by light gray), but in the case of Database 3, it did not recognize TP and FN, so balanced accuracy and sensitivity were undefined. The mean value of balanced accuracy is 100 ± 0 for Database 1, and 100 ± 0 for Database 2. MCC as the value that summarized the overall performance of the classifier is 1±0 for Database 1 and Database 2. kNN achieved the best performance for Database 3, the mean value of balanced accuracy is 98.56 ± 0.02, and MCC value 0.94 ± 0.08. ANN (marked by dark gray) has been shown as the most consistent classifier for the three datasets, regardless of the quality of the datasets and hardware realization acquisition system.

## 4. Conclusions

The main contribution of the study is the development of an ML-based method for detecting stress levels during driving, which has met the criteria for application in pervasive healthcare systems. The method consists of a pre-processing step, extraction of only four features, and classification by an artificial neural network. The proposed method is adapted to cECG signals recorded in unobtrusive acquisition systems, over clothes in a comfortable environment that enables everyday application. The feature list is formed based on two criteria: possible reliable detection of characteristic points regardless of quality recorded time series (recording conditions are harsher than clinical recordings, due to measurements over clothes and the movement of the driver while driving) and computationally non-demanding features to enable the real-time application. The proposed method is validated on three open datasets with different quality of recordings, due to the use of different hardware realization for the acquisition system of cECG and different conditions of recordings. High performances are achieved in terms of balanced accuracy and MCC for Database 1 (direct skin contact recordings), for Database 2 (non-direct skin contact, measurement through clothes by capacitive electrodes built-in portable cushion), and for Database 3 (non-direct skin contact, measurement through clothes by capacitive electrodes built-in car seat). The Matlab code of the proposed method is available per request.

In the future, the proposed model can be an integral part of an automatic diagnostic system based on unobtrusive recorded cECG, following the proposals from [39,40,41], which were developed for ECG recorded in controlled clinical conditions.

## Figures and Tables

**Table 1:** Absolute amplitude of cECG [mV], expressed as a mean ± standard deviation SD.

Database	Measurements	Hardware Realization	$fs\;\left[Hz\right]$	Number of Participants	Time Duration of Recording	cECG_1_	cECG_2_	cECG_3_
Database 1	direct skin contact		496	16	22.04 h	0.14 ± 0.17		
Database 2	non-direct skin contact	portable multi-modal cushion	128	20	8.1 h	6.24 ± 3.32		
Database 3	non-direct skin contact	Car seat	200	31	13.4 h	0.19 ± 0.67	0.29 ± 0.89	0.21 ± 0.69

**Table 2:** Classifier performances for ECG from Database 1 (direct skin contact recording). Expressed as a mean ± standard deviation SD.

Database 1 (Direct Skin Contact)	Balanced Accuracy	Sensitivity	Specificity	PPV	NPV	MCC
kNN	98.75± 2.5	97.5± 5	100± 0.00	100 ± 0.00	93.75 ±12.5	0.95 ± 0.09
SVM	100± 0	100± 0	100± 0	100± 0	100 ± 0	1 ± 0
ANN	100± 0	100± 0	100± 0	100± 0	100 ± 0	1 ± 0

**Table 3:** Classifier performances for cECG from Database 2 (non-direct skin contact, by sensors built in portable cushion). Expressed as a mean ± standard deviation SD.

Database 2 (Non-Direct Skin Contact, Portable Cushion)	Balanced Accuracy	Sensitivity	Specificity	PPV	NPV	MCC
kNN	94.88± 2.18	100± 0	89.76± 4.37	77.38 ± 8.41	100 ± 0	0.83 ± 0.06
SVM	100± 0	100±0	100± 0	100 ± 0	100 ± 0	1 ±0
ANN	96.67± 4.71	100± 0	93.33± 9.43	100 ±0	100 ± 0	0.89 ± 0.15

**Table 4:** Classifier performances for cECG from Database 3 (non-direct skin contact, by capacitive electrodes built-in car seat). Expressed as a mean ± standard deviation SD.

Database 3 (Non-Direct Skin Contact, Car Seat)	Balanced Accuracy	Sensitivity	Specificity	PPV	NPV	MCC
kNN	98.56± 0.02	100 ± 0	97.11± 3.68	90.97 ± 11.86	100 ± 0	0.94 ± 0.08
SVM	NaN	NaN	74.82± 0.93	0	100 ± 0	NaN
ANN	98.08± 0.02	100± 0	96.15± 3.85	87.96 ± 12.52	100 ± 0	0.91 ± 0.08

## Data Availability

Data is contained within the article. Datasets are available on UnoViS: The MedIT Public Unobtrusive Vital Sign Database. Available online: https://www.medit.hia.rwth-aachen.de/en/publications/unovis (accessed on 21 September 2023). and Stress Recognition in Automobile Drivers. Available online: https://physionet.org/content/drivedb/1.0.0/ (accessed on 21 September 2023).
